# Date of Publication of Matter Received

**Published:** 1913-03

**Authors:** 


### Date of Publication of Matter Received

A guest at a Thanksgiving dinner remarked to an author: "I suppose you are at work on a Christmas story now." "Well, scarcely," replied the author, "my Christmas stories were all sent in last spring and I am getting out a Fourth of July story now and am late at that." To prevent misunderstanding, the following obvious statements are published: The original articles for the JOURNAL are usually arranged three or four months in advance, though it is occasionally possible to publish a timely or perishable article received in the first few days of a month, in the next month's issue. Brief letters, news items, etc., can usually be published in the forthcoming issue if received by the fifteenth of the month. All matter is sent to the printer by the twentieth; in fact he usually has ten to twenty pages excess which is left in type for the second month after the current one; very brief personals, obituaries, and notes of special and immediate interest may be added as late as the twenty-fifth, crowding out Abstracts, New Inventions, Book Reviews, etc., to make space.

